# GDF-15 Suppresses Atherosclerosis by Inhibiting oxLDL-Induced Lipid Accumulation and Inflammation in Macrophages

**DOI:** 10.1155/2021/6497568

**Published:** 2021-09-07

**Authors:** Hong Huang, Zhongli Chen, Yan Li, Kunmei Gong, Le Xiao, Hao Fu, Jingjing Yang, Xianying Wang, Qiang Meng

**Affiliations:** ^1^Faculty of Life Science and Technology, Kunming University of Science and Technology, Kunming 650500, China; ^2^Department of Geriatric Medicine, The Affiliated Hospital of Kunming University of Science and Technology, The First People's Hospital of Yunnan Province, Kunming 650032, China; ^3^Department of Cardiology, Ruijin Hospital, Shanghai Jiaotong University, Shanghai 200025, China; ^4^Department of General Surgery, The First People's Hospital of Yunnan Province, Kunming 650032, China; ^5^Department of Neurology, The First People's Hospital of Yunnan Province, No. 157 Jinbi Road, Xishan District, Kunming 650032, Yunnan Province, China

## Abstract

The growth differentiation factor-15 (GDF-15) may be involved in atherosclerosis. However, the role of GDF-15 in atherosclerosis remains unclear. The main goal of this study was to verify the role and mechanism of GDF-15 in atherogenesis. We first compared the serum GDF-15 level between patients with coronary atherosclerosis and healthy people. And then one ApoE^−/−^ mouse model of atherosclerosis was used to explore the effects of GDF-15 on oxidized low-density lipoprotein (oxLDL) accumulation, atherosclerosis-related gene expression, and lipid accumulation-related protein expression in mouse macrophages. As a result, the level of serum GDF-15 in patients with coronary atherosclerosis was significantly higher than that in healthy people. In the mouse model, GDF-15 expression was elevated in the core of plaque, and it was secreted mainly by the macrophages. In addition, GDF-15 decreased oxLDL-induced lipid accumulation and inflammation activation in macrophages. GDF-15 decreased the mRNA expressions of CD36, LOX1, and TLR4 that are associated with lipoprotein accumulation in macrophages. Further study showed that GDF-15 might suppress oxLDL-induced lipoprotein accumulation via inhibiting CD36 and LOX1 and decrease inflammation in macrophages by inhibiting TLR4. Thus, GDF-15 may suppress atherosclerosis and plaque formation by inhibiting lipoprotein accumulation and inflammation activation.

## 1. Introduction

Atherosclerosis is the leading cause of vascular diseases, including ischemic heart disease, ischemic stroke, and peripheral arterial disease [1]. As a chronic inflammatory disorder, atherosclerosis usually happens at the inner curvatures and branch points of arterial vessels, where endothelium was activated by the laminar flows [2]. Once activated, endothelium increases its permeability to lipoproteins, leading to the accumulation of lipoprotein in the arterial vessel wall. The sequestered lipoproteins induce an inflammatory response, and then circulating monocytes are recruited to this site and differentiate into macrophages that can ingest sequestered lipoproteins, thus fighting against inflammation. However, this process cannot clear lipoproteins but transforms macrophages to lipoprotein-laden foam cells, which can secrete various substances to initiate and promote atherosclerotic plaque formation. During the formation of plaque, proinflammatory cytokines and chemokines released by macrophages and other cells play a crucial role [3].

GDF-15 belongs to the transforming growth factor-*β* (TGF-*β*)/bone morphogenetic proteins (BMPs) superfamily [4], which are potent regulators of vascular development and vascular remodeling and play a key role in atherosclerosis and restenosis. TGF-*β* and BMPs regulate the proliferation, differentiation, and survival of endothelial cells, smooth muscle cells, macrophages, and T cells through activating Smad-dependent and Smad-independent signaling via heteromeric type I and II receptor complexes and also participate in the response of vascular calcification cells [5]. Previous studies showed that GDF-15 expression was associated with the initiation and progression of multiple diseases [6]. For example, GDF-15 could induce the apoptosis of tumor cells to suppress the progression, invasiveness, and metastasis of tumors [7]. In addition, GDF-15 was reported to be correlated with cardiovascular and noncardiovascular mortality, and it played important roles in multiple cardiovascular diseases, such as heart failure, cardiac hypertrophy, and coronary heart disease [8–11]. A study of the atherosclerotic mouse model showed that GDF-15 increased the progress of the disease, and the expression defect of GDF-15 reduced the formation of early plaque and improved the stability of plaque [12]. Because the stability of plaque plays a blocking effect on the development of atherosclerosis and the deficiency of GDF-15 expression can improve the stability of plaque, some studies believe that the lack of GDF-15 is conducive to the body's resistance to vascular injury and inflammation [12, 13]. However, some other studies suggested that GDF-15 is a protective factor of the heart, which has the effect of antiatherosclerosis [14, 15]. It was reported that GDF-15 could inhibit apoptosis of endothelial cells induced by high glucose and improve the function of endothelial cells by activating PI3K/Akt/eNOS pathway [16]. Previous studies suggested GDF-15 might play an important role in inhibiting the occurrence of atherosclerosis, but the concrete role and mechanism of GDF-15 in atherosclerosis remain unclear.

In this study, we aimed to explore the role of GDF-15 in lipoprotein accumulation and inflammatory response in atherosclerosis. The serum GDF-15 level in atherosclerosis patients and healthy people and the expression and location of GDF-15 in the aorta atheromatous plaque in mice were detected first. Then the effect of GDF-15 on lipoprotein accumulation and proinflammatory cytokines releasing in oxidized low-density lipoprotein (oxLDL) treated macrophages was revealed. Further, we investigated the might mechanism of GDF-15 in oxLDL-induced lipoprotein accumulation and inflammation in macrophages in order to provide new clues for further understanding the role and mechanism of GDF-15 in atherosclerosis.

## 2. Materials and Methods

### 2.1. Human Sample Collection and Ethics Statement

This study involved the use of serum samples from healthy people and patients with atherosclerosis. Sixty-five health people and 101 patients were recruited in the First People's Hospital of Yunnan Province. After drawing blood, serum was isolated from collected blood and stored at −80°C until subsequent analysis. All samples were analyzed anonymously, and all researchers in this study have no access to any subject identification information. In addition, all recruited participants agree to the blood draw and signed written informed consents.

Ethical approval for the study and the informed consent process were approved by the Ethics Committee of the First People's Hospital of Yunnan Province (No. YYLH048). The research was conducted in accordance with the basic principles of the Helsinki declaration and the relevant international rules.

### 2.2. Enzyme-Linked Immunosorbent Assay (ELISA)

GDF-15 in human serum and interleukin-6 (IL-6), interleukin-8 (IL-8), monocyte chemoattractant protein-1 (MCP-1), and matrix metalloproteinase-9 (MMP-9) in the cell culture supernatant and mice serum were measured using ELISA kits according to manufacturers' protocol (ELISA Genie, Ireland).

### 2.3. Mouse Model of Atherosclerosis

Wild-type (WT) C57BL/6 mice and ApoE^−/−^ mice with a C57BL/6 background were purchased from the Model Animal Research Center of Nanjing University (Nanjing, China). The six-week-old C57BL/6 mice and ApoE^−/−^ mice were fed a high-fat diet (KlibaNafag 3200 supplemented with 1.25% w/w cholesterol and 15% w/w cacao butter) for 20 weeks and subsequently sacrificed. For further study, recombinant GDF-15 (50 mg/kg/d) was intravenously injected into ApoE^−/−^ mice after 10 weeks of high-fat feed once every three days. Aorta with plaques was collected at the end of 20 weeks of high-fat feed for subsequent H&E staining and immunohistochemical analysis. All animal experiments were approved by the Animal Ethics Committee of the First People's Hospital of Yunnan Province (Approval No. YYLH048).

### 2.4. Cell Culture and Treatment

Primary culture and identification of macrophages were derived from mouse bone marrow: the 6–8 week old mice were sacrificed and the femurs and tibias were taken. The bone marrow was placed in a 10 ml centrifuge tube. After lysis with RBC lysate, the supernatant was removed after centrifuged at 1,000 r/min for 5 min. The bone marrow cells were resuspended in the culture medium and inoculated evenly in a 10 cm culture dish. The cells were cultured overnight to remove other heterogeneous cells, such as fiber cells. The cells not attached to the wall were inoculated into a new culture plate and cultured with 50 ng/mL recombinant mouse M-CSF. On the third and seventh days, the activity and morphology of the cells were observed under an inverted microscope, including the adherent state, volume, morphology, and pseudopodia changes (photo recording). The macrophages from bone marrow were produced (the composition of the culture medium was *α*-MEM + 10% FBS + 1% double antibody). F4/80 and CD11 antibodies were used as a double standard to identify the purity of macrophages.

To study the effect of GDF-15 on macrophage functions, the macrophage was treated with oxLDL (50 *μ*g/mL) or oxLDL (50 *μ*g/mL) + GDF-15 (2 ng/mL) for a continuous 48 hours without changing the culture medium or other interruption. Cells and culture supernatant were collected at 48 hours post-treatment for the subsequent study. Then to verify the potential mechanism of the inhibitory effect of lipoprotein accumulation and inflammation in oxLDL-induced macrophage by GDF-15, overexpression vectors of CD36 and LOX1 (pcDNA-CD36 and pcDNA-LOX1, respectively) were constructed by Guangzhou RiboBio Biotechnology Co. Ltd. (Guangzhou, China) and transfected into macrophage cells with Lipofectamine® 2000 transfection reagent (Thermo Fisher Scientific, Inc., USA) following the instructions. And agonist of TLR4, Neoseptin-3 (20 *μ*M), was used to activate TLR4 in macrophage cells.

### 2.5. Oil Red O Staining

To evaluate the level of lipid accumulation in macrophages, the oil red O staining of macrophages was performed. Briefly, cells were fixed by 4% paraformaldehyde (PFA) for 15 min after being washed with PBS 3 times (5 min/time), then incubated with oil red O working solution (oil red:distilled water = 3:2) at room temperature for 10–15 min, subsequently differentiated by 60% isopropanol for 30 s, and washed with distilled water for 1 min. Finally, the filter paper was used to absorb the surrounding water, and glycerin gelatin was used to seal the cells. As a result, the lipid droplets are orange red to bright red stained.

### 2.6. Hematoxylin and Eosin and Nissl Staining

To verify whether atherosclerosis occurs in WT and ApoE^−/−^ mice, the aorta tissues were harvested, fixed with 4% PFA for 24 h, and dehydrated by washing with a series of ethanol solutions (75% ethanol for 2 h; 80% ethanol for 2 h; 95% ethanol I for 2 h; 95% ethanol II for 1.5 h; 100% ethanol I for 1 h; and 100% ethanol II for 30 min) at room temperature and then paraffin-embedded and sectioned (transverse section, 7 *μ*m thick). Then the sections were stained using the modified hematoxylin and eosin (H&E) staining kit (Solaria, China) in order to observe the morphological changes and plaques formation of aorta tissues. Images were captured under a light microscope (Nikon, Tokyo, Japan).

### 2.7. Immunohistochemistry Analysis

For immunohistochemistry analysis, the aorta tissue samples were fixed in 4% PFA for 24 h. Then the tissue block was put into the paraffin and sectioned into 7 *μ*m thick sections in a microtome. The section frame was put into the antigen repair solution and heated for 10 min under low fire. The slicing frame was taken out and put into PBS for 5 min; it was repeated 3 times. After washing, the samples were prepared for blocking and incubating with the primary antibodies of GDF-15 or IL-6 at 4°C overnight. Isotype-matched IgG was used instead of the primary antibody as a negative control of the staining. Sections were then incubated with diluted streptavidin-peroxidase HRP at room temperature with DAB color- and hematoxylin-rendering staining kit, following the manufacturer's instructions. Finally, it was sealed with neutral gum after dehydrating and drying by gradient alcohol. The sections were then stained with hematoxylin for 5 min and mounted and observed with a phase-contrast microscope.

### 2.8. Immunofluorescence Analysis

Fresh tissue samples were taken and immediately immersed in 4% PFA fixative solution, changed into 30% sucrose solution for 24 h, transferred into OCT glue after 4°C for 48 h, froze at −80°C for 30 min, and then sliced. Next, wash with PBST for 3 times, 5 min each time, at room temperature. Add 200 *μ*L sealing solution (10% goat serum) to the slice, and drop one prepared antibody (diluted with 10% goat serum, diluted according to the antibody manual, usually 1:200), and leave overnight to stay at 4°C. Wash three times with PBST, 5 min each time, add fluorescent second antibody, (1:2000) and incubate in dark for 1h; wash three times with 0.1% PBT, 5 min each time, at room temperature. DAPI was not exposed to light. Observe under the fluorescence microscope, and record the experimental results.

### 2.9. RNA Extraction and RT-qPCR Analysis

Total RNA was extracted from the macrophage using TRIzol (Ambion, USA) according to the manufacturer's protocol. Gene expression was quantified using iTaq Universal SYBR Green Supermix (Bio-Rad) and the CFX Connect real-time PCR detection system (Bio-Rad) after reverse transcription from RNA into cDNA using iScript reverse transcription supermix for RT-qPCR (Bio-Rad). Primers were designed to amplify the target genes. Primer sequences for gene expression analysis were shown in Table 1. The relative quantity of gene expression was calculated using the 2^−ΔΔCt^ method.

### 2.10. Western Blot Analysis

The macrophages were collected and lysed with RIPA buffer (Solarbio, China). Protein concentration was quantified using a Bio-Rad protein assay kit (Bio-Rad). Protein samples were separated by 12% SDS-PAGE. Then electrophoretically transferred onto polyvinylidene difluoride (PVDF) membranes (Bio-Rad). The membranes were incubated in primary antibodies (*β*-actin (Proteintech, China); IL-6 antibody; MCP-1 antibody; MMP-9 antibody, dilution 1:1,000 (CST, Germany); and IL-8 antibody, dilution 1:1,000 (Abcam, USA)) at 4°C overnight. Then membranes were incubated with horseradish peroxidase-coupled secondary antibody (antirabbit or antimouse antibodies, dilution 1:2,000 (Proteintech, China)) for 1 h at room temperature (Santa Cruz Biotechnology). The signals were detected by the chemiluminescence detection kit (Thermo Fisher Scientific). The protein bands were visualized by autoradiography and quantified by ImageJ software (National Institutes of Health, Bethesda, MD, USA).

### 2.11. Statistical Analyses

All results were confirmed in at least three independent experiments, and the data from one representative experiment was shown. All quantitative data are presented as mean ± SEM. The analysis was performed using GraphPad Prism version 7 (GraphPad Software, La Jolla, CA, USA). Statistical comparisons between each group were conducted with unpaired Student's *t*-test or one-way analysis of variance (ANOVA) followed by Bonferroni's multiple comparisons test. For all comparisons, *P* < 0.05 was considered to indicate a statistically significant difference.

## 3. Results

### 3.1. Elevated Expression of GDF-15 in Atherosclerosis Patients

**Figure 1:**
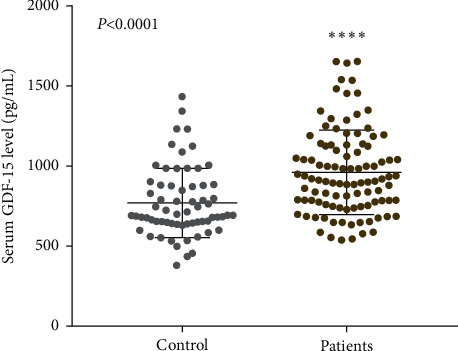
Level of serum GDF-15 in patients with atherosclerosis. Serum GDF-15 levels in patients with atherosclerosis were detected and compared with that of healthy people. Results showed the level of GDF-15 in patients was significantly higher than that in healthy controls. ^*∗∗∗∗*^*P* < 0.0001 vs. control group.

One previous atherosclerosis mouse model study demonstrated that GDF-15 deficiency attenuates early atherogenesis and improves plaque stability [12]. The clinical study suggested that a high level of GDF-15 is associated with coronary artery disease, a disease involving chronic inflammatory atherosclerosis [17]. To explore the relationship of GDF-15 with atherosclerosis, we recruited 65 healthy people and 101 patients with coronary atherosclerosis. After the blood draw, serum was isolated to measure GDF-15. As a result, GDF-15 levels were significantly higher in patients than that in healthy controls (960.1 ± 26.35 pg/mL vs. 769.6 ± 26.75 pg/mL; *P* < 0.0001, as can be seen in Figure 1) detected by ELISA assay. This finding indicates that the serum GDF-15 level may be associated with atherosclerosis.

### 3.2. High Expression of GDF-15 in Mice Macrophages with Atherosclerosis

**Figure 2:**
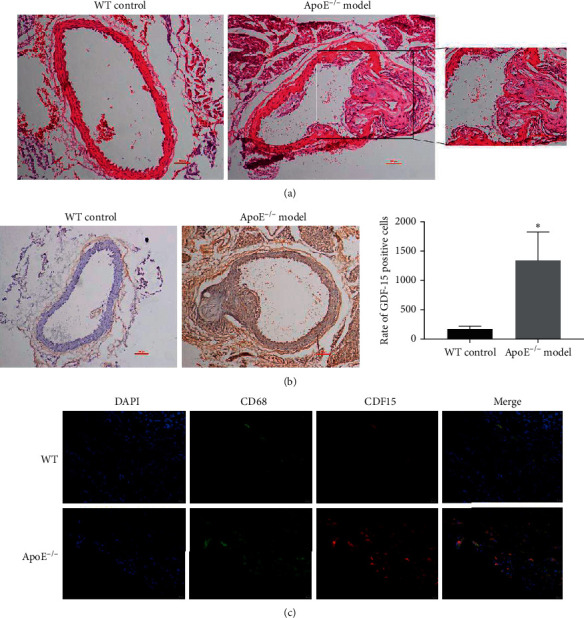
Macrophages are the major GDF-15 secreting cells in atherosclerotic plaque. (a) H&E staining. After fed by high-fat diet, the endothelium of the artery in ApoE^−/−^ mice was damaged obviously, accompanied with the formation of atherosclerotic plaque, while there was no obvious change in wild-type mice artery. (b) GDF-15 was highly expressed in blood vessels of ApoE^−/−^ mice compared with that in wild-type mice detected by immunohistochemical analyses. (c) Immunofluorescence staining showed most of GDF-15 were produced by CD68 positive macrophages, which suggested that macrophages may be the major cells, which producing GDF-15.

To further determine the correlation of GDF-15 with atherosclerosis, we compared GDF-15 expression in macrophages residing in blood vessels. Herein, the ApoE^−/−^ mice model of atherosclerosis was used, and blood vessels with an atherosclerotic plaque were studied. As shown in Figure 2(a), compared with wild-type mice, ApoE^−/−^ mice fed by a high-fat diet showed significant aortic arch stenosis, accompanied by obvious endothelial cell damage and atherosclerotic plaque formation. Meanwhile, immunohistochemical staining showed that the expression of GDF-15 in the aorta of ApoE^−/−^ mice with atherosclerotic plaque was significantly higher than that of WT mice (Figure 2(b)), which is consistent with the expression of serum GDF-15 (Figure 1). Furthermore, immunofluorescence staining results showed that the level of CD68 positive macrophages and expression of GDF-15 in arterial tissue of WT mice were lower, while the positive rates of CD68 and GDF-15 in ApoE^−/−^ mice were significantly higher than those in WT mice, and most GDF-15 producing cells were CD68 positive macrophages (Figure 2(c)), which suggested that macrophages may be the main cells producing GDF-15. Thus, our data suggested that the expression of GDF-15 was upregulated in atherosclerosis produced mainly by macrophages.

### 3.3. GDF-15 Suppressed Lipid Accumulation in Macrophages

**Figure 3:**
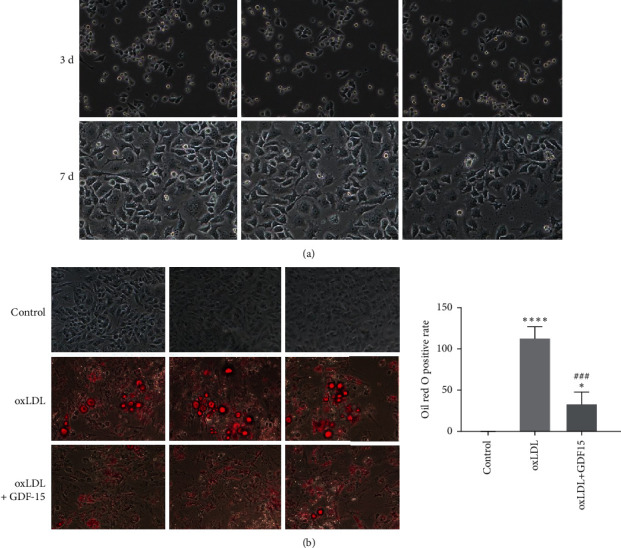
GDF-15 suppressed lipoprotein accumulation in oxLDL-treated monocyte-derived macrophages (MDMs): (a) representative images of primary cultured MDMs cells and (b) oil red O staining showed oxLDL treatment significantly induced lipoprotein accumulation in MDMs, while GDF-15 treatment inhibited oxLDL-induced lipoprotein accumulation. All images were obtained using a ZEISS LSM confocal microscope. Values are expressed as the mean ± standard deviation of three independent experiments as determined by Student's *t*-test. ^*∗*^*P* < 0.05, ^*∗∗∗∗*^*P* < 0.0001 vs. control group; ^###^*P* < 0.001 vs. oxLDL-induced group.

Macrophages under endothelial cells play a decisive role in atherosclerosis initiation and progression [2, 18]. Macrophages in atherosclerotic lesions actively participate in lipoprotein ingestion and accumulation, which contributes to lipoprotein storage under endothelial cells and atherosclerosis initiation and progression [19]. To explore the role of GDF-15 in atherogenesis, we first induced primary culture macrophages, monocyte-derived macrophages (MDMs; Figure 3(a)), and studied the effect of GDF-15 on lipoprotein storage in macrophages by oil red O staining. As a result, oxLDL treatment alone induced lipoprotein accumulation in macrophages, while GDF-15 treatment significantly suppressed oxLDL-induced lipoprotein accumulation (Figure 3(b)). These results suggested that GDF-15 reduced lipoprotein accumulation in macrophages.

### 3.4. GDF-15 Suppressed Inflammation Response in Macrophages

**Figure 4:**
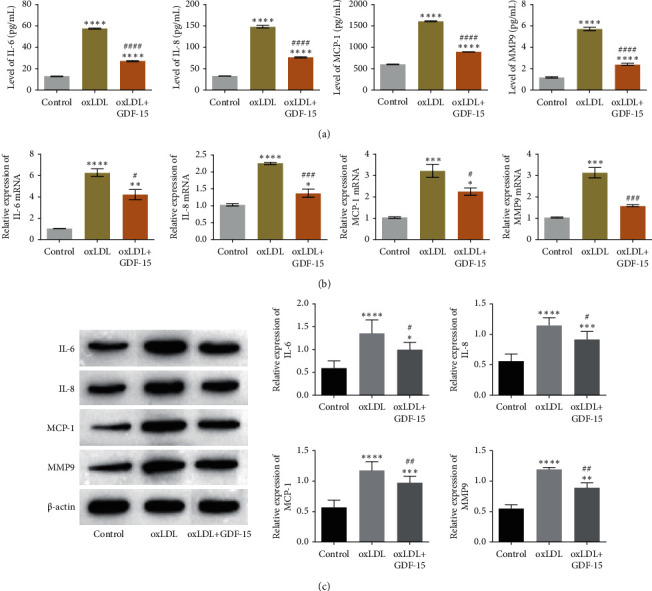
GDF-15 suppressed oxLDL-induced proinflammatory cytokine productions in MDMs. (a) The results of ELISA assay showed that oxLDL treatment promoted MDMs secret IL-6, IL-8, MCP-1, and MMP9, while GDF-15 inhibited oxLDL-induced proinflammatory cytokines releasing in MDMs. (b) After treated by oxLDL, the mRNA levels of IL-6, IL-8, MCP-1, and MMP-9 were significantly upregulated but then suppressed by GDF-15 treatment. (c) The protein levels of IL-6, IL-8, MCP-1, and MMP-9 were confirmed by western blotting assay, and the results showed that the protein levels of these proinflammatory cytokines in oxLDL-induced MDMs were obviously downregulated by GDF-15 treatment. Values are expressed as the mean ± standard deviation of three independent experiments as determined by one-way ANOVA test. ^*∗∗*^*P* < 0.01, ^*∗∗∗*^*P* < 0.001 vs. control group; ^#^*P* < 0.05,  ^##^*P* < 0.01,  ^###^*P* < 0.001 vs. oxLDL-induced group.

The inflammation response is a remarkable feature of atherosclerosis, and oxLDL has been demonstrated to trigger inflammation response and proinflammatory cytokine production [3]. To determine the effect of GDF-15 on atherogenesis, we studied the effect of GDF-15 on proinflammatory cytokines, chemokines, and matrix protein, which all are correlated with atherosclerosis initiation and development in the presence of oxLDL. Consistent with a previous study [3], our results showed that oxLDL treatment upregulated the secretions of IL-6, IL-8, MCP-1, MMP-9, and GDF-15 treatment obviously suppressed the oxLDL-induced release of the above-studied proteins (Figure 4(a)). Next, to explore the mechanism that GDF-15 inhibits protein secretion, we performed RT-qPCR assay and western blot assay to study the effect of GDF-15 on transcription and protein expression of intracellular proteins. As a result, oxLDL treatment upregulated the intracellular expression of IL-6, IL-8, MCP-1, and MMP-9 in both mRNA and protein levels, while GDF-15 treatment suppressed the intracellular mRNAs and protein expressions of these proinflammatory cytokines (Figures 4(b) and 4(c)). Thus, the above data suggested that GDF-15 could inhibit the oxLDL-induced inflammatory response, which promotes atherosclerosis initiation and development.

### 3.5. GDF-15 Decreased Gene Expressions That Are Associated with Lipoprotein Accumulation

**Figure 5:**
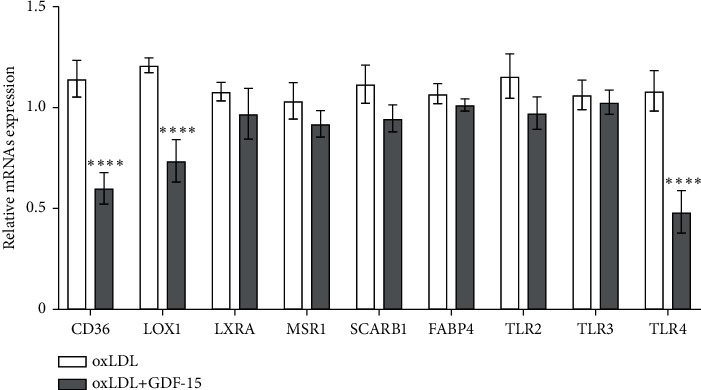
The effect of GDF-15 on gene expression associated with lipoprotein accumulation. oxLDL-induced MDMs were treated with GDF-15 (2 ng/mL) or untreated for 48 hours. After treatment, the mRNA expressions of CD36, LOX1, LXRA, MSR1, SCARB1, FABP4, TLR2, TLR3, and TLR4 were confirmed by real-time PCR assay. The results showed that the mRNA expressions of CD36, LOX1, and TLR4 were obviously downregulated by GDF-15 in oxLDL-induced MDMs. ^*∗∗∗∗*^*P* < 0.0001 vs. oxLDL-induced group.

Subsequently, we explored the potential mechanism that GDF-15 blocks atherogenesis. Lipoprotein accumulation in macrophages is central to the pathogenesis of atherosclerosis and one of the earliest events in plaque formation [20]. Herein, we measured the impact of GDF-15 on gene expressions that are related to lipoprotein accumulation. The macrophages were treated with oxLDL (50 *μ*g/mL) or oxLDL (50 *μ*g/mL) + GDF-15 (2 ng/mL) for 48 hours. After treatment, the CD36, LOX1, LXRA, MSR1, SCARB1, FABP4, TLR2, TLR3, and TLR4 mRNA levels were measured by RT-qPCR assay. The results showed that GDF-15 significantly decreased levels of three genes, CD36, LOX1, and TLR4 (Figure 5), in oxLDL-induced macrophages. This result indicates that CD36, LOX1, and TLR4 might be involved in GDF-15-mediated lipoprotein accumulation suppression.

### 3.6. GDF-15 Suppressed oxLDL-Induced Lipoprotein Accumulation via Inhibiting DC36 and LOX1

**Figure 6:**
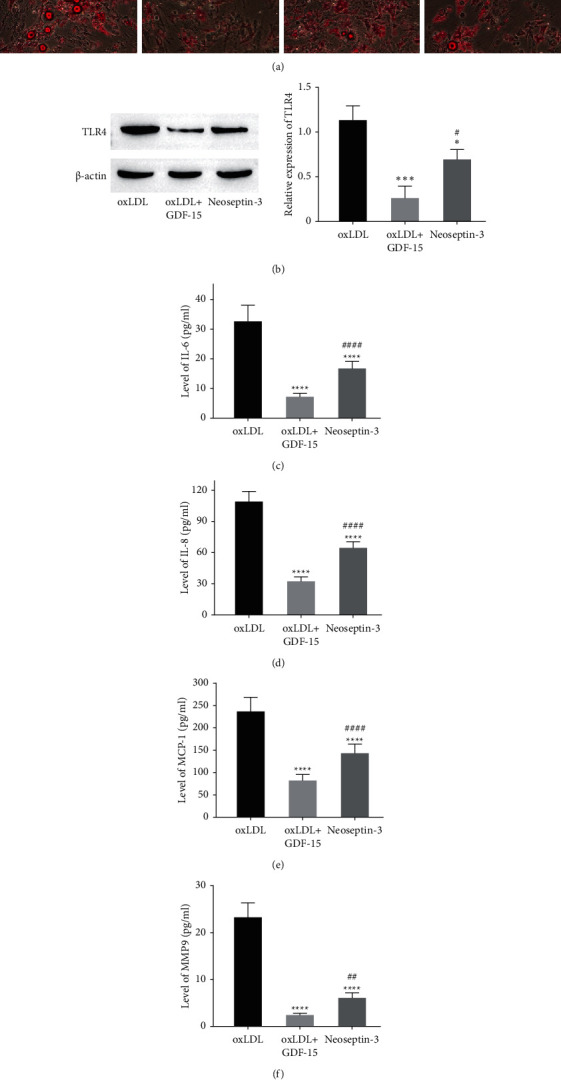
GDF-15 suppressed oxLDL-induced lipoprotein accumulation via inhibiting DC36 and LOX1. (a) Oil red O staining showed that GDF-15 inhibited lipoprotein accumulation in oxLDL-induced MDMs, but overexpression of both CD36 and LOX1 reversed the effect of GDF-15 treatment. (b) GDF-15 downregulated the expression of TLR4 induced by MDMs, while Neoseptin-3 reversed the inhibitory effect of GDF-15. (c–f) The levels of IL-6, IL-8, MCP-1, and MMP9 in oxLDL-induced MDMs were significantly suppressed by GDF-15, while Neoseptin-3 treatment reversed the levels of these proinflammatory cytokines. Values are expressed as the mean ± standard deviation of three independent experiments as determined by one-way ANOVA test. ^*∗*^*P* < 0.05,  ^*∗∗∗*^*P* < 0.001,  ^*∗∗∗∗*^*P* < 0.0001 vs. oxLDL-induced group; ^#^*P* < 0.05,  ^##^*P* < 0.01,  ^####^*P* < 0.0001 vs. oxLDL + GDF-15-treated group.

To determine whether GDF-15 inhibits oxLDL-induced lipid accumulation in macrophages, Transfect pcDNA‐CD36 and pcDNA‐LOX1 into MDMs cells to overexpress CD36 and LOX1, then induce untransfected and transfected MDMs cells with oxLDL, and treat them with GDF15 for 48 hours. Oil red O staining showed GDF‐15‐inhibited lipid accumulation in oxLDL‐induced MDMs, but overexpression of both CD36 and LOX1 reversed the effect of GDF‐15 treatment (Figure 6(a)), which indicated that GDF-15 suppressed oxLDL-induced lipid accumulation partly by inhibiting CD36 and LOX1. Then, to verify whether GDF-15 inhibits oxLDL-induced macrophages inflammation via inhibiting TLR4, Neoseptin-3, an agonist of TLR4, was used to activate TLR4 when the oxLDL-induced cells were treated by GDF-15. The results showed that GDF-15 downregulated the expression of TLR4 induced by MDMs, while Neoseptin-3 reversed the inhibitory effect of GDF-15 (Figure 6(b)). Furthermore, the levels of IL-6, IL-8, MCP-1, and MMP9 in oxLDL-induced MDMs were significantly suppressed by GDF-15, while Neoseptin-3 treatment reversed the levels of these proinflammatory cytokines (Figures 6(c)–6(f)), which indicated that GDF-15 decreases proinflammatory cytokines releasing in oxLDL-induced macrophages by inhibiting the activation of TLR4 signaling.

### 3.7. Effect of Recombinant GDF-15 on High-Fat Fed ApoE^−/−^ Mice

**Figure 7:**
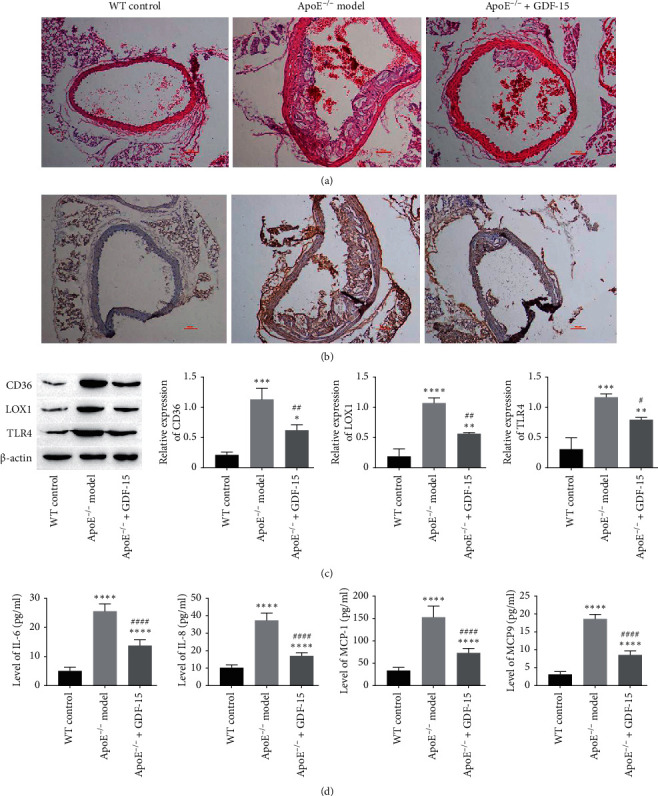
Effect of recombinant GDF-15 on high-fat fed ApoE^−/−^ mice: (a) representative H&E staining images of artery vessels in WT mice, ApoE^−/−^ mice, and recombinant GDF-15-treated ApoE^−/−^ mice; (b) representative IL-6 immunohistochemical staining images of artery vessels in WT mice, ApoE^−/−^ mice, and recombinant GDF-15-treated ApoE^−/−^ mice; (c) expressions of CD36, LOX1, and TLR4 in artery vessels of WT mice, ApoE^−/−^ mice, and recombinant GDF-15-treated ApoE^−/−^ mice; and (d) levels of IL-6, IL-8, MCP-1, and MMP9 in artery vessels of each group. Values are expressed as the mean ± standard deviation of three independent experiments of five independent animal samples as determined by one-way ANOVA test. ^*∗∗*^*P* < 0.01,  ^*∗∗∗*^*P* < 0.001,  ^*∗∗∗∗*^*P* < 0.0001 vs. WT control group; ^#^*P* < 0.05,  ^##^*P* < 0.01,  ^####^*P* < 0.0001 vs. ApoE^−/−^ model group.

To determine the effect of recombinant GDF-15 on the inflammatory response and atherosclerotic plaque formation of ApoE^−/−^ mice, recombinant GDF-15 was an intravenous injection to upregulate the level of circulating GDF-15. As the results showed, recombinant GDF-15 obviously alleviated the endothelial cell injury and atherosclerotic plaque formation in the aorta of ApoE^−/−^ mice (Figure 7(a)). Immunohistochemistry staining of IL-6 showed that IL-6 was highly expressed in the plaque of ApoE^−/−^ mice aortic arch, while recombinant GDF-15 decreased the positive rate of IL-6 in the plaque of ApoE^−/−^ mice aorta (Figure 7(b)). Further study showed that GDF-15 could inhibit the expression of CD36, LOX1, and TLR4 in the aorta of ApoE^−/−^ mice (Figure 7(c)) and decrease the levels of IL-6, IL-8, MCP-1, and MMP9 in peripheral blood of ApoE^−/−^ mice (Figure 7(d)). These results indicated that intravenous injection of recombinant GDF-15 could ameliorate atherosclerosis.

## 4. Discussion

GDF-15 is a new member of the transforming growth factor beta (TGF-*β*) superfamily, which has most recently been found in activated macrophages. GDF-15 is inducible in human macrophages by oxLDL and its mediators in vitro and is supposed to contribute to oxidative stress-dependent consequences in arteriosclerotic plaques modulating apoptosis and inflammatory processes in activated macrophages [21]. To determine the relationship of GDF-15 with atherosclerosis, we compared the serum GDF-15 levels in patients with atherosclerosis and healthy people. As a result, GDF-15 was highly expressed in patients compared with healthy people (Figure 1). We also measured GDF-15 in atherosclerosis plaques from the ApoE^−/−^ mice model of atherosclerosis. In mice with atherosclerosis, GDF-15 was highly expressed in the core of plaque and macrophages (Figure 2(b)). This study suggested that GDF-15 is possibly associated with atherosclerosis and can play a protective role.

Atherosclerosis is initiated by lipoprotein accumulation in the intimal layer of the arterial vessel wall, which results in the recruitment of circulating monocytes [22]. Once entered the arterial intima, the monocytes differentiate into macrophages; then the latter ingests lipoproteins, in particular oxidized lipoproteins, and finally converts into foam cells, which promotes atherosclerosis progression [23–26]. To determine the real role of GDF-15 in atherosclerosis, we detected the effect of GDF-15 on lipoprotein accumulation and inflammatory response in oxLDL-induced macrophages. Lipoprotein accumulation analysis showed that GDF-15 suppressed lipid accumulation in oxLDL-treated macrophages (Figure 3). Meanwhile, GDF-15 contributed to a decreased inflammatory response in oxLDL-treated macrophages (Figure 4). These data suggested that GDF-15 could suppress atherosclerosis initiation and progression. In this study, a GDF-15 inhibitor was not used to confirm the effect of GDF-15 on lipid accumulation and inflammatory factors, which can become future research directions.

Inflammation response plays an important role in the development and progression of atherosclerosis. Proinflammatory cytokine IL-6 is a proatherogenic cytokine, and sustained IL-6 production promotes the development of atherosclerosis [27–29]. Recombinant IL-6 treatment exacerbates atherosclerosis in wild-type and atherosclerosis-prone ApoE^−/−^ mice fed with a high-fat diet, which shows a dramatically increased lesion size [26]. Another proinflammation cytokine, IL-8, and chemokine, MCP-1, are involved in monocyte adhesion and migration into the arterial vessel wall in atherosclerosis [29]. MMP belongs to a very large family of peptidases that can degrade the extracellular matrix [30]. MMP-9 is a member of the MMP family, and it has been determined to contribute to the destabilization of the plaque [3]. This study showed that GDF-15 could decrease the levels of IL-6, IL-8, MCP-1, and MMP-9 in oxLDL-treated macrophages (Figure 4). This result suggested that GDF-15 has pluripotential atheroprotective effects, that is, blocking the entry of monocytes into the arterial wall and suppressing plaque formation and expansion.

To study the atheroprotective mechanism of GDF-15, we studied the effect of GDF-15 on gene expressions that are associated with lipoprotein accumulation in macrophages. As a result, we found GDF-15 could decrease the expressions of CD36, LOX1, and TLR4 (Figure 5). Both CD36 and LOX1 are scavenged receptors, which recognize and process modified LDL (such as oxLDL) [31]. Previous studies indicate that CD36 locating in the surface of macrophages can bind oxLDL and then promotes oxLDL internalization [32]. On the other hand, the interaction of CD36 with oxLDL can also activate the immune response and then result in the secretion of cytokines, which lead to immune cell infiltrates and finally promote the progress of atherosclerosis [33]. Like CD36, LOX1 can also bind oxLDL, and ApoE^−/−^ mouse model research indicated that LOX1 overexpression dramatically enhanced atherosclerosis [34]. This study showed that GDF-15 suppressed the expression of CD36 and LOX1, and GDF-15 might suppress oxLDL-induced lipoprotein accumulation by inhibiting the expression of CD36 and LOX1 (Figure 6(a)), but the concrete molecular mechanism needs further study. On the other hand, GDF-15 treatment also decreased TLR4 expression. Previous studies suggested that TLR4 plays a role in atherosclerosis. As a pattern recognition receptor, TLR4 activation can induce a proinflammatory response and then enhance atherogenesis. Moreover, minimally, oxLDL can be recognized by TLR4 and promotes TNF-*α* and IL-6 production [35]. Thus, our results showed that GDF-15 suppressed proinflammatory response induced by oxLDL and attenuated atherosclerosis initiation as well as progression by downregulating TLR4 (Figures 6(b) and 6(c)).

In conclusion, this study showed that GDF-15 suppressed atherosclerosis by inhibiting lipid accumulation and decreasing the expressions of IL-6, IL-8, MCP-1, and MMP-9 in macrophages. Furthermore, the expression of CD36, LOX1, and TLR4 may play an important role in the atheroprotective mechanism of GDF-15. However, the concrete molecular mechanism needs further exploration. This study indicated that GDF-15 may suppress atherosclerosis and plaque formation by inhibiting lipoprotein accumulation and inflammation activation. Although the results of this study are inconsistent with some existing studies, and the role of GDF-15 in atherosclerosis is still controversial, which needs further research and elucidation, GDF-15 still has the potential to become a target for the diagnosis and treatment of atherosclerosis.

## Figures and Tables

**Table 1:** The sequences of primers used in real-time PCR.

Gene name	Product size (bp)	Number gene primer (5′–3′)
*β*-actin	224	F: TGCTGTCCCTGTATGCCTCT
		R: TTTGATGTCACGCACGATTT
CD36	96	F: GGTGATGAGAAGGCAAAC
		R: CACCACACCAACACTGAG
LOX1	173	F: GGATGCCAAGTTGCTGA
		R: CGCCTCGGACTCTAAATA
LXRA	151	F: GTTTGCCTTGCTCATTGC
		R: TGGGAACATCAGTCGGTC
MSR1	143	F: CACTGATTGCCCTTTACC
		R: TCCCGTGAGACTTTGAG
SCARB1	187	F: CAGGGAGTTCAGGCACAAA
		R: CTTCAGGGTCATGGGCTTA
FABP4	102	F: TGGGATGGAAAATCAACC
		R: TCTCTCATAAACTCTCGTGG
TLR2	222	F: GATGCCTACTGGGTGGA
		R: AAGACGGAAATGGGAGA
TLR3	70	F: CAACGACTGATGCTCCGAAG
		R: GAAGAGGCTGGAATGGTGAA
TLR4	287	F: GGTATTTGACACCCTCCAT
		R: TTCTGTTCCTTGACCCACT
IL-6	256	F: GTGTCTTTCCCGTGGACCTTC
		R: TCATCTCGGAGCCTGTAGTGC
IL-8	140	F: CCTGGAAATCAACAGTTATGCT
		R: AGGTTCAGCAGGTAGACATCG
MCP-1	125	F: TCCTGTCATTTATGCCTTTGTTG
		R: CACTCGATCTGCTGTCTCCCTAT
MMP-9	260	F: CCCACTTACTATGGAAACTCAA
		R: CTCAAAGATGAACGGGAACA

## Data Availability

The data sets used and/or analyzed during the current study are available from the corresponding author upon reasonable request.
